# Immediate versus Delayed Sarcoma Reconstruction: Impact on Outcomes

**DOI:** 10.1155/2016/7972318

**Published:** 2016-07-13

**Authors:** Kyle J. Sanniec, Cristine S. Velazco, Lyndsey A. Bryant, Nan Zhang, William J. Casey III, Raman C. Mahabir, Alanna M. Rebecca

**Affiliations:** ^1^Department of Plastic Surgery, University of Texas Southwestern, 1801 Inwood Road, Dallas, TX 75390, USA; ^2^Division of General Surgery, Mayo Clinic in Arizona, 5777 E. Mayo Boulevard, Phoenix, AZ 85054, USA; ^3^Division of Plastic and Reconstructive Surgery, Mayo Clinic in Arizona, 5777 E. Mayo Boulevard, Phoenix, AZ 85054, USA; ^4^Division of Health Sciences Research, Mayo Clinic in Arizona, 13400 East Shea Boulevard, Scottsdale, AZ 85259, USA

## Abstract

*Background.* Sarcoma is a rare malignancy, and more recent management algorithms emphasize a multidisciplinary approach and limb salvage, which has resulted in an increase in overall survival and limb preservation. However, limb salvage has resulted in a higher rate of wound complications.* Objective.* To compare the complications between immediate and delayed (>three weeks) reconstruction in the multidisciplinary limb salvage sarcoma patient population.* Methods.* A ten-year retrospective review of patients who underwent sarcoma resection was performed. The outcome of interest was wound complication in the postoperative period based on timing of reconstruction. We defined infection as any infection requiring intravenous antibiotics, partial flap failure as any flap requiring a debridement or revision, hematoma/seroma as any hematoma/seroma requiring drainage, and wound dehiscence as a wound that was not completely intact by three weeks postoperatively.* Results.* 70 (17 delayed, 53 immediate) patients who underwent sarcoma resection and reconstruction met the inclusion criteria. Delayed reconstruction significantly increased the incidence of postoperative wound infection and wound dehiscence. There was no difference in partial or total flap loss, hematoma, or seroma between the two groups.* Discussion and Conclusion.* Immediate reconstruction results in decreased wound complications may reduce the morbidity associated with multidisciplinary treatment in the limb salvage sarcoma patient.

## 1. Introduction

Sarcoma remains a rare malignancy and accounts for less than 1% of all newly diagnosed cancers (~11,000 new diagnoses a year in the United States) [1]. While early descriptions of treatment were focused on limb amputation, more recent management algorithms emphasize a multidisciplinary approach and limb salvage when feasible. The result of this evolution in care is increased overall survival and limb preservation [2], but the tradeoff being a higher rate of wound complications [3–5]. In exploring the etiology of these wound complications, our group uncovered a potential benefit in immediate reconstruction following sarcoma resection [6]. In an effort to further clarify the effect, we designed this study to compare the complications between immediate and delayed reconstruction in the limb salvage sarcoma patient population.

## 2. Methods

With IRB approval, a ten-year retrospective review of all patients who underwent sarcoma resection at our institution was performed. Patients with defects closed primarily were excluded from the study. The intervention compared was the timing of the reconstruction: delayed versus immediate. Delayed reconstruction was defined as any reconstruction occurring three weeks after primary oncologic resection, and immediate reconstruction was defined as any reconstruction within a three-week timeframe. Three weeks was chosen as the delayed reconstruction period, as patients had their first postoperative visit with orthopedics following oncologic resection; if wound complications were noted at that time, plastic and reconstructive surgery was consulted. Minimal dressings and debridement were used in the intervening time frame prior to delayed reconstruction. Demographic information and patient comorbidities, including obesity, peripheral vascular disease, coronary artery disease, diabetes, and active smoking or alcohol abuse, were collected. Neoadjuvant and intraoperative radiation and respective dosing were also recorded. The primary outcome of interest to the multidisciplinary team was wound complication in the postoperative period. To minimize observer, reporting, and recall bias, we defined infection as any infection requiring intravenous antibiotics, partial flap failure as any flap requiring a debridement or revision, hematoma/seroma as any hematoma/seroma requiring drainage, and wound dehiscence as a wound that was not completely intact by three weeks postoperatively.

## 3. Statistical Analysis

Patients' demographics, diagnosis, chemotherapy, radiation, and additional health problems were summarized overall and compared between delayed and immediate reconstruction groups. Fisher's exact test was used for categorical variable comparisons, and two-sample *t*-test was used for continuous variable comparisons. Fisher's exact test was used to compare the outcomes between delayed and immediate reconstruction groups.

## 4. Results

A total of 70 (17 delayed, 53 immediate) patients who underwent sarcoma resection and reconstruction met the inclusion criteria. The most common pathologic diagnosis was myxofibrosarcoma in both reconstructive groups; for complete details see Table 1. The median age of the patient population was 66.5 (range: 17–94) years, and 62.9% were female. Obesity was the only significant comorbidity (*p* = 0.006) (Table 1). For type of flap, refer to Table 2.

Radiation was delivered to both groups (Table 1). 82.4% of the delayed group received neoadjuvant radiation, whereas 64.2% of the immediate group did (*p* = 0.2324); of those patients who received radiation, median dose was 50.4 gy and 50.0 gy (*p* = 0.3955), respectively. 52.9% of the delayed group received intraoperative radiation, whereas 30.2% of the immediate group did (*p* = 0.1443); of those patients who received intraoperative radiation, median dose was 12.5 gy in both groups (*p* = 0.5157).

Among patients who had delayed reconstruction, there were significantly more patients with wound infections (*n* = 8, 47.1%) and wound dehiscence (*n* = 11, 64.7%) compared to patients who had immediate reconstruction (wound infection: *n* = 5, 9.4%, *p* = 0.0016; wound dehiscence: *n* = 16, 30.2%, *p* = 0.0203). There was no difference between partial or total flap failure, hematoma, and seroma (Table 3).

## 5. Discussion

The reconstruction of sarcoma defects continues to represent a perfect storm for the plastic surgeon: a large tissue defect in a radiated field in a patient who has undergone chemotherapy. The most conspicuous finding of this study is that immediate reconstruction appears to result in a decreased rate of wound complications. This supports our previous work [6] on the importance of early plastic surgical intervention and reconstruction. Nearly 50% of the patients undergoing delayed reconstruction were diagnosed with an infection requiring intravenous antibiotics. Immediate reconstruction with vascularized soft tissue introduces healthy, nonradiated tissue, increases local blood flow, increases bacterial clearance, and consequently decreases infection rates.

Previous studies [7, 8] have demonstrated an infection rate from 15 to 30% in sarcoma patients, and in our study infection was seen in 47% of delayed reconstruction patients. This would suggest that there might be a role for prophylactic antibiotics for sarcoma patients who are undergoing delayed reconstruction. Interestingly, there are no current guidelines for prophylactic antibiotic use in this setting. Our results may also provide insight for those patients in whom immediate reconstruction is not an option.

There was no significant difference when evaluating partial or total flap loss between reconstructive groups. However, the only flap loss seen was in the immediate reconstructive group. While flap failure rate was low (5.7%), we hypothesize that this may be due to the defect size caused by oncologic resection. Orthopedic surgeons may have more readily involved the reconstructive team when planning for a large resection that would require flap coverage. Lohman et al. observed this as well when comparing primary versus flap reconstruction. They hypothesize that filling the dead space created by the defect with well vascularized tissue may prevent wound complications when tissue pliability is lost from radiation or the immune system is compromised from comorbidities such as diabetes [9].

Neoadjuvant radiation can be associated with increased wound complications compared to postoperative radiation, which has more long-term morbidity and worse functional outcomes. However, these wound complications are often acute and manageable following neoadjuvant therapy [10]. Additionally, neoadjuvant therapy has been shown to have wound complication rates of 32% [11], and in a randomized trial, Baldini et al. found an overall complication rate of 52% following neoadjuvant radiation therapy among patients that required flap reconstruction [12]. Immediate reconstruction with vascularized tissue may provide improved wound healing through increased oxygenation and enhance antibacterial activity following radiation [13]. Our current work supports that decreased rates of wound complications are seen in immediate reconstruction following radiation.

The current study is a retrospective review and as such has limitations, including observer and selection bias. In an effort to minimize the effect of observer bias, we determined the outcomes of interest to be as objectively measured as possible. Initially, the orthopedic surgeons were performing the resections, intraoperative radiation, and the primary closure. As plastic surgeons made themselves available in sarcoma reconstruction, our group began doing more immediate reconstructions. The decrease in intraoperative radiation may be due to the ability to perform wider resection margins knowing that vascularized closure was readily available. While it would be a better study design to perform a prospective trial, ethical standards do not allow for this type of evaluation.

## 6. Conclusion

Delayed reconstruction had a significantly higher incidence of infection and wound dehiscence when compared to immediate reconstruction in the sarcoma limb salvage patient population. Immediate reconstruction may reduce the morbidity associated with this complex reconstruction.

## Figures and Tables

**Table 1:** Characteristics of the study population by reconstructive type.

	Delayed (*N* = 17)	Immediate (*N* = 53)	Total (*N* = 70)	*p* value^1^
*Age*				0.3613
Mean (SD)	59.8 (16.9)	64.3 (18.0)	63.2 (17.8)	
Median (range)	66.0 (17.0–81.0)	67.0 (22.0–94.0)	66.5 (17.0–94.0)	

*Sex, female (%)*	11 (64.7%)	33 (62.3%)	44 (62.9%)	1.0000

*Diagnosis, n*				0.4740
Myxofibrosarcoma	5 (29.4%)	7 (13.2%)	12 (17.1%)	
Synovial sarcoma	1 (5.9%)	5 (9.4%)	6 (8.6%)	
Leiomyosarcoma	2 (11.8%)	4 (7.5%)	6 (8.6%)	
Liposarcoma	2 (11.8%)	3 (5.7%)	5 (7.1%)	
Sarcoma NOS	2 (11.8%)	4 (7.5%)	6 (8.6%)	
Myxoid chondrosarcoma	1 (5.9%)	0 (0.0%)	1 (1.4%)	
Myxoid liposarcoma	2 (11.8%)	2 (3.8%)	4 (5.7%)	
Fibrous histiocytoma	0 (0.0%)	5 (9.4%)	5 (7.1%)	
Fibrosarcoma	0 (0.0%)	4 (7.5%)	4 (5.7%)	
Pleomorphic sarcoma	0 (0.0%)	7 (13.2%)	7 (10.0%)	
Spindle cell sarcoma	1 (5.9%)	3 (5.7%)	4 (5.7%)	
Pleomorphic liposarcoma	1 (5.9%)	0 (0.0%)	1 (1.4%)	
Dermatofibrosarcoma protuberans	0 (0.0%)	1 (1.9%)	1 (1.4%)	
Angiosarcoma, high grade	0 (0.0%)	1 (1.9%)	1 (1.4%)	
Giant cell rich extraosseous osteosarcoma	0 (0.0%)	1 (1.9%)	1 (1.4%)	
Fibroblastic sarcoma	0 (0.0%)	1 (1.9%)	1 (1.4%)	
Epithelioid angiosarcoma	0 (0.0%)	2 (3.8%)	2 (2.9%)	
Osteosarcoma	0 (0.0%)	1 (1.9%)	1 (1.4%)	
Neurofibrosarcoma	0 (0.0%)	1 (1.9%)	1 (1.4%)	
Epitheloid sarcoma	0 (0.0%)	1 (1.9%)	1 (1.4%)	

*Neoadjuvant chemotherapy, n *	8 (47.1%)	14 (26.4%)	22 (31.4%)	0.1381

*Neoadjuvant radiation, n*	14 (82.4%)	34 (64.2%)	48 (68.6%)	0.2324

*Neoadjuvant radiation dosage, gy *				0.1167
Mean (SD)	39.9 (19.7)	30.0 (23.3)	32.4 (22.7)	
Median	50.0 (0.0–50.4)	45.0 (0.0–50.4)	50.0 (0.0–50.4)	

*Intraoperative radiation, n *	9 (52.9%)	16 (30.2%)	25 (35.7%)	0.1443

*Intraoperative radiation dosage, gy *				0.1240
Mean (SD)	6.8 (6.7)	4.0 (6.2)	4.7 (6.4)	
Median (Range)	10.0 (0.0–15.0)	0.0 (0.0–17.5)	0.0 (0.0–17.5)	

*Obesity, n*	8 (47.1%)	7 (13.2%)	15 (21.4%)	0.0060

*Peripheral Vascular Disease, n*	0 (0.0%)	1 (1.9%)	1 (1.4%)	1.0000

*Coronary Artery Disease, n*	1 (5.9%)	5 (9.4%)	6 (8.6%)	1.0000

*Diabetes, n*	4 (23.5%)	4 (7.5%)	8 (11.4%)	0.0911

*Current smoker, n*	1 (5.9%)	5 (9.4%)	6 (8.6%)	1.0000

*Alcohol abuse, n*	0 (0.0%)	1 (1.9%)	1 (1.4%)	1.0000

^1^Fisher's exact test was used for categorical variables and two sample *t*-test was used for continuous variables. (*p* < 0.05 significant). gy = gray.

**Table 2:** Flap type.

Type of flap	Delayed (*N* = 17)	Immediate (*N* = 53)	Total (*N* = 70)
Free TRAM	4 (23.5%)	3 (5.7%)	7 (10.0%)
Pedicle TRAM	3 (17.6%)	4 (7.5%)	7 (10.0%)
Free VRAM	1 (5.9%)	0 (0.0%)	1 (1.4%)
Pedicle VRAM	1 (5.9%)	4 (7.5%)	5 (7.1%)
Pedicle ALT	3 (17.6%)	0 (0.0%)	3 (4.3%)
Free ALT	0 (0.0%)	3 (5.7%)	3 (4.3%)
Pedicle rectus abdominis	3 (17.6%)	0 (0.0%)	3 (4.3%)
Free rectus abdominis	0 (0.0%)	2 (3.8%)	2 (2.9%)
Free gracilis	1 (5.9%)	0 (0.0%)	1 (1.4%)
Pedicle gracilis	0 (0.0%)	1 (1.9%)	1 (1.4%)
Pedicle gastrocnemius	1 (5.9%)	5 (9.4%)	6 (8.6%)
STSG	0 (0.0%)	5 (9.4%)	5 (7.1%)
FTSG	0 (0.0%)	2 (3.8%)	2 (2.9%)
Pectoralis major/latissimus dorsi	0 (0.0%)	1 (1.9%)	1 (1.4%)
DIEP	0 (0.0%)	1 (1.9%)	1 (1.4%)
Free serratus anterior	0 (0.0%)	1 (1.9%)	1 (1.4%)
TAP	0 (0.0%)	1 (1.9%)	1 (1.4%)
Free latissimus	0 (0.0%)	3 (5.7%)	3 (4.3%)
Pedicle latissimus	0 (0.0%)	2 (3.8%)	2 (2.9%)
Reverse radial forearm	0 (0.0%)	3 (5.7%)	3 (4.3%)
Pedicle TFL	0 (0.0%)	1 (1.9%)	1 (1.4%)
Fasciocutaneous advancement	0 (0.0%)	1 (1.9%)	1 (1.4%)
Pedicle rectus femoris	0 (0.0%)	3 (5.7%)	3 (4.3%)
Sural nerve graft	0 (0.0%)	1 (1.9%)	1 (1.4%)
Free fasciocutaneous	0 (0.0%)	1 (1.9%)	1 (1.4%)
Pedicle fasciocutaneous	0 (0.0%)	1 (1.9%)	1 (1.4%)
Local rotational	0 (0.0%)	1 (1.9%)	1 (1.4%)
Free lateral arm	0 (0.0%)	2 (3.8%)	2 (2.9%)
Hemisoleus	0 (0.0%)	1 (1.9%)	1 (1.4%)

TRAM = transverse rectus abdominis myocutaneous; VRAM = vertical rectus abdominis myocutaneous; STSG = split thickness skin graft; ALT = anterolateral thigh; FTSG = full thickness skin graft; RF = rectus femoris; TFL = tensor fascia lata; DIEP = deep inferior epigastric artery perforator.

**Table 3:** Outcomes by reconstructive type (univariate).

Outcome	Delayed (*N* = 17)	Immediate (*N* = 53)	*p* value^1^
Infection requiring IV antibiotics	8 (47.1%)	5 (9.4%)	0.0016
Flap failure	0 (0.0%)	3 (5.7%)	1.0000
Partial flap loss	4 (23.5%)	5 (9.4%)	0.2059
Wound dehiscence/drainage	11 (64.7%)	16 (30.2%)	0.0203
Hematoma	4 (23.5%)	3 (5.7%)	0.0542
Seroma	5 (29.4%)	11 (20.8%)	0.5133

^1^ *p* value is from Fisher's exact test.
